# Clinical and Molecular Biomarkers for Diagnosis and Staging of NAFLD

**DOI:** 10.3390/ijms222111905

**Published:** 2021-11-02

**Authors:** Stefania Di Mauro, Alessandra Scamporrino, Agnese Filippello, Antonino Di Pino, Roberto Scicali, Roberta Malaguarnera, Francesco Purrello, Salvatore Piro

**Affiliations:** 1 Department of Clinical and Experimental Medicine, Internal Medicine, Garibaldi-Nesima Hospital, University of Catania, 95122 Catania, Italy; 8stefaniadimauro6@gmail.com (S.D.M.); alessandraska@hotmail.com (A.S.); agnese.filippello@gmail.com (A.F.); antonino.dipino@unict.it (A.D.P.); robertoscicali@gmail.com (R.S.); salvatore.piro@unict.it (S.P.); 2 Faculty of Medicine and Surgery, “Kore” University of Enna, 94100 Enna, Italy; roberta.malaguarnera@unikore.it

**Keywords:** biomarkers, ncRNAs, NAFLD

## Abstract

Non-alcoholic fatty liver disease (NAFLD) is the most common hepatic pathology in industrialized countries, affecting about 25% of the general population. NAFLD is a benign condition, however, it could evolve toward more serious diseases, including non-alcoholic steatohepatitis (NASH), fibrosis, cirrhosis, and finally, hepatocellular carcinoma (HCC). Liver biopsy is still the gold standard for NAFLD diagnosis. Due to the risks associated with liver biopsy and the impossibility to apply it on a large scale, it is now necessary to identify non-invasive biomarkers, which may reliably identify patients at higher risk of progression. Therefore, several lines of research have tried to address this issue by identifying novel biomarkers using omics approaches, including lipidomics, metabolomics and RNA molecules’ profiling. Thus, in this review, we firstly report the conventional biomarkers used in clinical practice for NAFL and NASH diagnosis as well as fibrosis staging, and secondly, we pay attention to novel biomarkers discovered through omics approaches with a particular focus on RNA biomarkers (microRNAs, long-noncoding RNAs), showing promising diagnostic performance for NAFL/NASH diagnosis and fibrosis staging.

## 1. Introduction

Non-alcoholic fatty liver disease (NAFLD) is the most common hepatic pathology in industrialized countries, and it is associated with an increase in morbidity and mortality. NAFLD represents the hepatic manifestation of metabolic syndrome, which is considered a systemic disease since it affects different organs, including pancreas [1], kidney, adipose tissue and gut [2,3,4]. It is estimated that NAFLD affects about 25% of the general population [5], although the real prevalence is unknown because of the lack of specific and sensitive diagnostic tests. NAFLD includes several pathological conditions, ranging from simple steatosis to non-alcoholic steatohepatitis (NASH), the latter characterized by hepatic necroinflammation and rapid progression toward fibrosis, cirrhosis, and finally, hepatocellular carcinoma (HCC) [5,6,7,8]. NAFLD diagnosis is an exclusion diagnosis in which hepatic biopsy is still the gold standard to discriminate simple steatosis from NASH and to stage fibrosis. Over the last few years, the identification of non-invasive biomarkers to differentiate simple steatosis from NASH and stage fibrosis has become one of the most important aims of research in the hepatological field. This issue is very relevant because of the high prevalence of NAFLD in the general population, the potential progression toward more serious diseases and the impossibility to apply hepatic biopsy on a large scale.

**Figure 1:**
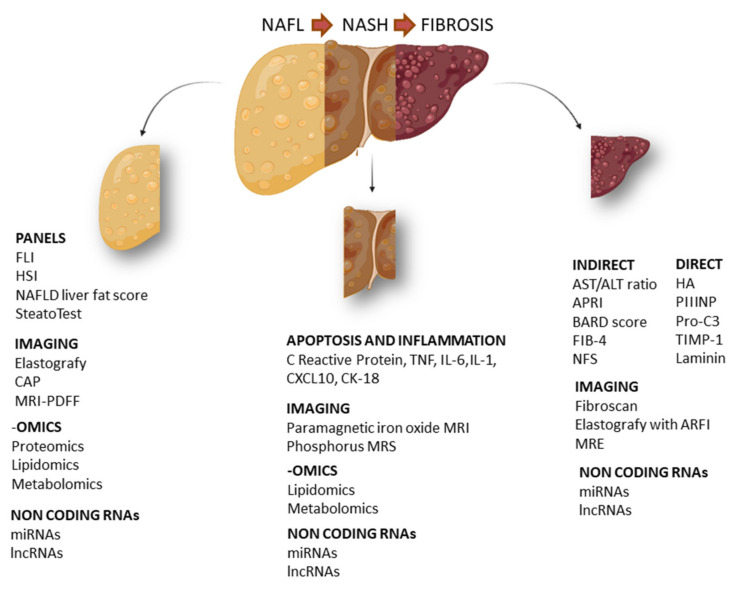
Schematic representation of the main circulating biomarkers in NAFLD. Specific biomarkers for NAFL (non-alcoholic fatty liver), NASH (non-alcoholic steatohepatitis) and hepatic fibrosis. FLI (fatty liver index), HSI (hepatic steatosis index), CAP (controlled attenuation parameter), MRI-PDFF (magnetic resonance imaging proton density fat fraction), TNF (tumor necrosis factor), IL-6 (interleukin 6), IL-1 (interleukin 1), CXCL10 (C-X-C motif chemokine ligand 10), CK18 (cytokeratin 18), APRI (AST to platelet ratio index), BARD score (BMI, AST:ALT ratio, presence of diabetes), FIB-4 (fibrosis 4), NFS (NAFLD fibrosis score), HA (hyaluronic acid), PIIINP (N-terminal type III collagen pro-peptide), Pro-C3 (C-terminal cleavage site of N-terminal type II collagen pro-peptide), TIMP-1 (tissue inhibitor of metalloproteinases 1), MRE (magnetic resonance elastography).

In this article, we aim to analyze both routine biomarkers currently used in clinical practice and novel biomarkers for the NAFLD spectrum, including non-alcoholic fatty liver (NAFL), NASH and fibrosis. In particular, we focus our dissertation on clinical biomarkers, their combination, imaging biomarkers and biomarkers identified through high-throughput approaches (Figure 1).

## 2. NAFL Biomarkers

NAFL diagnosis involves the determination of hepatic steatosis (defined as the histological presence of triglyceride accumulation in 5% of hepatocytes) and exclusion of other causes of hepatic damage in people not assuming or assuming limited amounts of alcohol < 20/30 g per day respectively, for women and men [9,10]. Several non-invasive methods attempting to replace hepatic biopsies for NAFL diagnosis have been investigated [11,12].

### 2.1. Serum Biomarkers and Relative Panels

During the last few years, several indexes and scores have been elaborated to diagnose hepatic steatosis [13,14]. FLI (fatty liver index) includes BMI, waist circumference, serum triglycerides and GGT (gamma-glutamyl transferase). FLI has a moderate diagnostic performance (AUC = 0.84) in discriminating the presence of lipids in the liver as well as echographic analysis [15]; however, it has low accuracy in identifying several grades of steatosis [16]. HSI (hepatic steatosis index) is a panel of biomarkers that includes ALT/AST, BMI, sex and presence of type 2 diabetes, and this index has a moderate diagnostic power for hepatic steatosis identification [17] that considerably decreases in obese children (AUC = 0.67) [18]. Furthermore, similarly to FLI, it has a low accuracy in identifying several grades of hepatic steatosis [16]. A more efficient and sensitive method is the NAFLD liver fat score; through magnetic resonance, this method determines liver fat and combines different parameters, including the presence of metabolic syndrome and type 2 diabetes, insulin level, AST and ALT/AST. This score has a good diagnostic performance to diagnose hepatic steatosis with an AUC of 0.87 [19], however, the inclusion of insulin level, which is not a routinely used test, limits its use in clinical practice. SteatoTest is a panel including more specific parameters to diagnose steatosis. It combines six FibroTest elements (α2-macroglobulin, haptoglobin, apolipoprotein A1, GGT, total bilirubin and ALT) in addition to BMI, cholesterol, triglycerides and glycemia. SteatoTest is adjusted for sex and age, but it has a moderate accuracy to predict hepatic steatosis confirmed by hepatic biopsy (AUC = 0.80) [20]. This test is not widely used because it is expensive and is not able to discriminate different levels of steatosis.

### 2.2. Imaging Biomarkers

Imaging methods are often used in clinical practice and are used for NAFL diagnosis. The most used imaging method is echography, which is not expensive and is easy to perform. A meta-analysis demonstrated that elastography has high accuracy in discriminating moderate from severe steatosis (AUC = 0.93) [21]. However, this method has some limits: it is not able to detect steatosis when it is less than 20%, it is influenced by the presence of fibrosis and it is operator-dependent [11]. CAP (controlled attenuation parameter) detects the grade of steatosis, measuring the level of ultrasound attenuation due to the presence of fat. It has been reported that CAP has an AUC of 0.82 in detecting every grade of steatosis versus the absence of steatosis [22]. CAP has some limits, including a low sensibility for the low grade of steatosis, and it is operator-dependent. Magnetic resonance imaging proton density fat fraction (MRI-PDFF) is a non-invasive method that maps liver fat. MRI-PDFF is more precise than CAP in detecting several steatosis grades in NAFLD patients (AUC = 0.99) [23]; however, it is time-consuming, very expensive and needs qualified personnel.

### 2.3. Omics-Based Biomarkers

**Figure 2:**
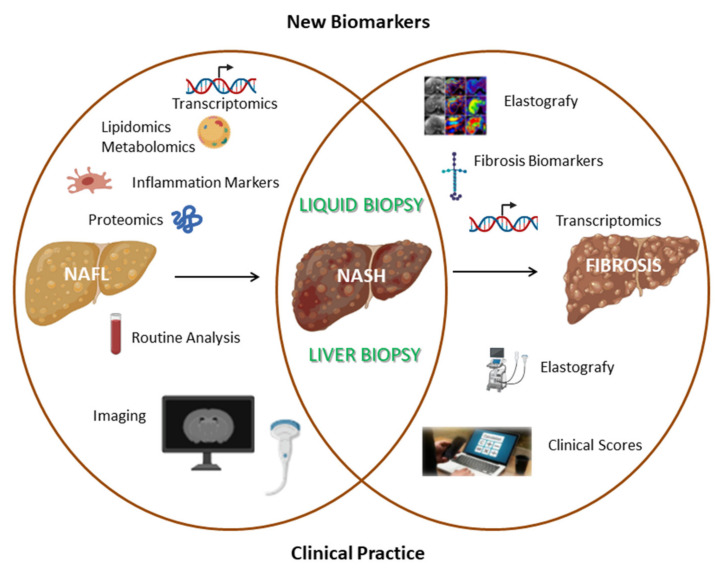
Comparison between commonly used clinical methods and innovative research fields aimed at identifying novel non-invasive biomarkers for simple steatosis, NASH and fibrosis biomarkers.

“Omics technologies”, through the detection of thousands of different molecules, can identify novel biomarkers that could be useful for NAFL diagnosis. Proteomics simultaneously analyzes a wide range of proteins by using a small volume of biological fluids. A study conducted on 70 patients (35 controls and 35 NAFL) identified 20 protein peaks in NAFL versus control (sensitivity 89% and specificity 83%); furthermore, through this approach, it has been reported that NAFL patients had a higher basal hemoglobin level [24]. In another study, through a more sensitive proteomic technique (LFQP), 605 differentially expressed proteins were observed in NAFL patients, however this method failed in discriminating simple steatosis patients with respect to NASH [25]. Metabolomics has been applied to identify the metabolic profile specific for NAFL, such as bile acids and glutathione, whose levels are altered during NAFLD onset [26]. Lipidomic-based studies identify the alteration of lipid species levels, for instance, short-chain fatty acids and eicosanoids [13]. A study performed on 679 patients reported that three specific lipids (defined as “lipid triplet”) can identify NAFL, however the diagnostic performance is limited (AUC = 0.71–0.74) [27]. Omics-based biomarkers could also be applied to evaluate disease severity; for instance, a study conducted on 467 NAFLD patients identified a signature of triglycerides that was able to differentiate NAFL from NASH (AUROC = 0.79) [28]. However, these methods have some drawbacks: a validation cohort is often lacking, the patient sample size is limited, and they often fail in discriminating several grades of steatosis and disease progression (Figure 2).

## 3. NASH Biomarkers

NASH patients have a higher probability of developing cirrhosis and of dying from cardiovascular or liver-related causes [29,30]. Therefore, it is extremely important to identify NASH and fibrosis patients early among NAFLD patients in order to reduce the mortality. Thus, several lines of research attempted to develop non-invasive tests to discriminate NAFL from NASH. To achieve this aim, they mainly focused on biochemical and molecular biomarkers involved in specific pathways of progression from NAFL to NASH. Signaling pathways related to NASH development include apoptosis, oxidative stress, inflammation networks and adiponectin-mediated signals.

### 3.1. Protein Serum and Plasma Biomarkers Associated with Apoptosis and Inflammation

Apoptosis plays a fundamental role in the hepatic damage observed in NASH, and cytokeratin-18 (CK-18) is a biomarker of this process. CK-18 is indeed the main protein constituting hepatocyte intermediate filaments. When these cells undergo to apoptosis, CK-18 is cleaved by caspase 3 and CK-18 fragments are released at the extracellular level [31]. Serum CK-18 fragment levels are easily measurable through the ELISA assay. A meta-analysis of 11 studies highlighted that CK-18 has an overall sensitivity of 66% and an overall specificity of 82%, and therefore the limited accuracy of this biomarker prevents its clinical use for screening analysis and NASH definition [32]. CK-18 has been combined with other indicators in a biomarker panel aiming to increase the diagnostic performance for NASH patient identification. M30 serum levels deriving from CK-18 have been added to the quantification of the serum apoptosis-mediating surface antigen sFAS, involved in the activation of the extrinsic pathway of apoptosis in hepatocytes. The combination of these two markers determined a considerable increase in diagnostic performance, reaching an AUC value of 0.79–0.93. However, this study was conducted on a small sample, and thus this result needs further validation [33]. Younossi et al., combining CK-18 and relative fragments with adiponectin and resistin dosage in a cohort of 101 patients, obtained AUC values between 0.73 and 0.91. This study was conducted on a small sample of obese patients, and therefore other validations are needed [34]. Another study demonstrated that the combination of fibroblast growth factor 12 (FGF12) and CK-18 values increases sensitivity and specificity to values higher than 90% [35].

Since steatohepatitis is characterized by a chronic inflammation state, several studies evaluated markers of inflammation, such as C-reactive protein (CPR), TNF, IL-6, IL-1, IL-1RA (receptor antagonist protein) and CXCL10 (C-X-C motif chemokine ligand 10). However, these studies obtained inconsistent and weak results and a lack of specificity for the NASH pathological condition [36]. Ajmera et al. analyzed the diagnostic performance of 32 plasmatic biomarkers, most of them associated with inflammation, in a cohort of 648 patients. Among the analyzed biomarkers, after clinical and metabolic parameters’ adjustment, only the activated plasminogen activator inhibitor (APAI-1) maintained its predictive power for NASH [37].

### 3.2. Omics-Based Biomarkers

Oxidative stress is one of the key mechanisms of hepatic damage, and leads to lipid oxidation products that can be detected in serum samples.

Lipidomics studies performed using mass spectrometry led to the identification of specific products deriving from fatty acids associated with NASH. In particular, arachidonic acid oxidation products, including 11-hydroxyeicosatetraenoic acid (11-HETE), and linoleic acid oxidation products such as hydroxyoctadecadienoic acid (HODE) and 9 and 13 oxo-octadecadienoic acids (oxo-ODE), have been identified as biomarkers of NASH. Linoleic acid and 13-HODE ratios have been added to other clinical parameters (age, BMI and AST) in order to construct the oxNASH panel. This panel has a sensitivity of 81% and specificity of 97% in NASH versus not NASH discrimination [38].

In another study performed through the metabolomics approach, an increase in serum taurine- and glycine-conjugated primary and secondary bile acids in NASH patients after feeding was observed [39].

### 3.3. Imaging Biomarkers

Routine imaging methods such as abdominal ultrasonography, CT or MRI are able to discriminate NAFL from NASH. Several techniques based on MRI have been tested in animals and in pilot studies conducted in humans for NASH diagnosis. The superparamagnetic iron oxide MRI can detect defective Kupffer cell uptake function in in vivo models and in humans affected by NASH. However, the current protocol involves repeated scanning over the course of 72 h, which represents a logistic limit of this method [40]. Hepatocyte membrane turnover and intracellular ATP that are altered in NASH can be detected through phosphorus MRS. In more detail, alpha-nucleotide triphosphate/total phosphate ratio has an AUC value of 0.71 for NASH patient discrimination [41]. Finally, the diagnostic performance of hepatic stiffness, measured through magnetic resonance elastography, needs to be evaluated in a larger cohort and adjusted for fibrosis stages [42] (Figure 2).

## 4. Fibrosis Biomarkers

NASH-related fibrosis includes different stages, ranging from absent fibrosis (F0) to cirrhosis (F4). From a clinical point of view, fibrosis is defined as clinically significant (F2–F4) or advanced/severe fibrosis (F3–F4). It is widely known that there are several risk factors that are able to predict fibrosis onset, including age, severe obesity, type 2 diabetes mellitus, high AST/ALT ratio, hypertension, dyslipidemia and the presence of the metabolic syndrome [43,44,45].

According to a study published in hepatology, the hepatic fibrosis stage could be the most important factor in determining NAFLD prognosis and preventing the risk of progression toward cirrhosis and related complications [46].

To stage fibrosis in NAFLD patients, it is important to identify novel non-invasive biomarkers. The most used biomarkers in clinical practice are classified into (a) indirect biomarkers of hepatic fibrosis, (b) direct biomarkers of hepatic fibrosis and (c) imaging biomarkers for fibrosis.

### 4.1. Indirect Biomarkers for Hepatic Fibrosis

Indirect biomarkers do not directly measure fibrogenesis or fibrinolysis, but are often associated with risk factors for fibrosis; furthermore, in order to increase their limited accuracy, two or more of them are combined in panels.

AAR (AST:ALT ratio) and APRI (AST:platelet ratio index) are the simplest indexes to calculate, but they have low accuracy in discriminating advanced stage 3 fibrosis (AUC AST:ALT ratio: between 0.66 and 0.74; AUC APRI: 0.74) [47,48]. The BARD score is based on the sum of three parameters (BMI, AST:ALT ratio, presence of diabetes), and it has a moderate accuracy in identifying F3 fibrosis subjects (AUC between 0.69 and 0.81) [49,50]. The FIB-4 index (fibrosis index based on four factors) involves a more complicated calculation, and thus online calculators are often used: it includes age, AST, ALT and platelet count, and it has a good diagnostic performance (AUC: 0.80 in identifying F3 fibrosis stage subjects) [51]. The NAFLD fibrosis score (NFS) [52] is obtained by combing age, presence of diabetes, body mass index, platelets, albumin and AAR. This score has been validated in several studies and it has an estimated AUC of between 0.75 and 0.83 in F3 fibrosis detection [53]. 

Although these scores have less accuracy than direct specific fibrosis markers, they are the most commonly used indexes for screening thanks to their wide applicability.

### 4.2. Fibrosis Biomarkers

Fibrosis biomarkers include molecules directly involved in fibrogenesis and/or fibrinolysis. Hepatic fibrosis is determined by the accumulation of extracellular matrix components, where hyaluronic acid (HA) is the most represented [54]. Serum levels of HA have an AUC of 0.87 for stage F2 fibrosis and 0.92 for cirrhosis [55]. Other components of the extracellular matrix include collagen, proteoglycans, elastin, fibronectin and laminin. Serum procollagen III amino-terminal peptide (PIIINP) originates from novel collagen III biosynthesis or existing collagen III fibril degradation. PIIINP levels alone represent a good diagnostic biomarker for fibrosis [56], but it has been demonstrated that Pro-C3 (the PIIINP neo-epitope) reflects the effective production of collagen III biosynthesis [57]; furthermore, the increase of serum Pro-C3 levels is correlated with NASH and fibrosis [58]. The circulating level of metalloprotease-1 inhibitor (TIMP1) influences extracellular matrix composition, wound healing and reflects the alteration of tissue matrix remodeling during hepatic fibrogenesis and fibrinolysis [59]. TIMP1 has excellent diagnostic performance (AUC: 0.97) in the discrimination of obese patients with NASH-related fibrosis with respect to matched controls [60]. Laminin is the most abundant glycoprotein in the basal membrane and its serum levels are able to identify the presence of fibrosis in NAFLD patients with excellent diagnostic performance (AUC: 0.87) [61].

The enhanced liver fibrosis test (ELF) is a panel analyzing direct biomarkers of fibrosis; specifically, it detects extracellular matrix components. ELF is based on the detection of three fibrosis biomarkers (HA, TIMP1 and PIIINP) and it has excellent diagnostic power in advanced fibrosis identification in adult and pediatric NAFLD patients (AUC 0.93 and 0.99, respectively) [62,63]. Another instrument for fibrosis detection is the FibroTest, that is obtained through the combination of five biomarkers (haptoglobin, α2-macroglobulin, Apolipoprotein A1, total bilirubin and GGT). The diagnostic value of the FibroTest has been evaluated in a wide cohort of NAFLD patients and reached an AUC of 0.88 [63].

The NAFLD FibroMeter is an index including weight, prothrombin index and serum level of ALT, AST, ferritin and fasting glucose. In two different studies conducted in Asia and Europe, the NAFLD FibroMeter surpassed the other serum test for F2 patient discrimination (AUC: 0.76) and F3 patient discrimination (AUC: 0.77) [64,65]. Finally, the Hepascore, composed of age, sex, gender, bilirubin serum level, GGT, HA and α2m, has a good diagnostic performance (AUC: 0.82) to diagnose advanced fibrosis (stages F3–F4) in NAFLD patients [66].

Although these indexes have a diagnostic performance superior to indirect biomarkers of fibrosis, their large-scale use is limited because of the reduced number of known direct fibrosis biomarkers, their cost and the use of a patented formula.

### 4.3. Imaging Biomarkers of Fibrosis

Over the last ten years, the progress in imaging methods improved the possibility to quantify, in a non-invasive manner, hepatic fibrosis, revolutionizing hepatic disease clinical management. The FibroScan or VTCE (vibration controlled transient elastography) is based on the use of an echographic probe that transmits a vibratory wave propagating through the liver, evaluating liver elasticity, and the velocity of wave propagation is directly proportional to tissue rigidity. Results are expressed in kilopascal (kPa). Usually, the mean value of ten measurements in healthy subjects is between 1.5 and 7.5 kPa, while values > 10.5 kPa are an index of the presence of fibrosis and advanced fibrosis [67]. Unfortunately, VCTE or M probes are less reliable in severe obese NAFLD patients, because adipose tissue attenuates both elastic waves and ultrasound [68]. Therefore, to overcome this issue, a novel probe “XL” has been developed for patients with BMI > 30 kg/m^2^. The diagnostic performances of FibroScan probes M and XL for advanced fibrosis are 0.88 and 0.85, respectively [69].

Elastography conducted through the ARFI method (acoustic radiation force impulse) is an alternative method to estimate hepatic tissue rigidity that can be integrated with a conventional ultrasound probe, detecting stiffness during routine echography. A region of interest is excited mechanically using acoustic impulses. Hepatic rigidity is expressed in m/s, and this method has a limited range (0.5–4.4 m/s) that limits the definition of a cut-off value to discriminate specific stages of fibrosis. It has been reported that the diagnostic accuracy of elastography with the ARFI technique is higher in discriminating severe fibrosis and cirrhosis (AUC: 0.74–0.97 for F3; AUC: 0.78–0.89 for F4) rather than less severe stages (AUC: 0.70–0.83 for F2) [70].

Magnetic resonance electrography (MRE) is another non-invasive technique to detect fibrosis in NAFLD patients. It combines magnetic resonance with a sound wave to create a visible map (elastogram) that shows hepatic tissue rigidity [71,72]. In more detail, a meta-analysis of 9 studies including 232 NAFLD patients highlighted that MRE can detect fibrosis with high accuracy, independently from hepatic inflammation, and BMI (differently from FibroScan) with an AUC of 0.86–0.91 for each stage of fibrosis [73]. This method has a higher accuracy than FibroScan in detecting F2 fibrosis (AUC: 0.86–0.89 versus AUC: 0.84) and F4 fibrosis (AUC: 0.88–0.97 versus AUC: 0.95) [74]. However, its large-scale application as a screening test is limited by cost and the small number of identified molecules (Figure 2).

## 5. RNA Biomarkers

In biological fluids, there are RNA molecules belonging to different classes, including messenger RNAs (mRNAs), long-noncoding RNAs (lncRNAs), circular RNAs (circRNAs) and microRNAs (miRNAs). The presence of cell-free RNAs in body fluids stimulated the interest in studying these molecules as novel biomarkers of diagnosis, prognosis, monitoring and therapeutic response of several kinds of pathologies [75,76,77,78].

**Figure 3:**
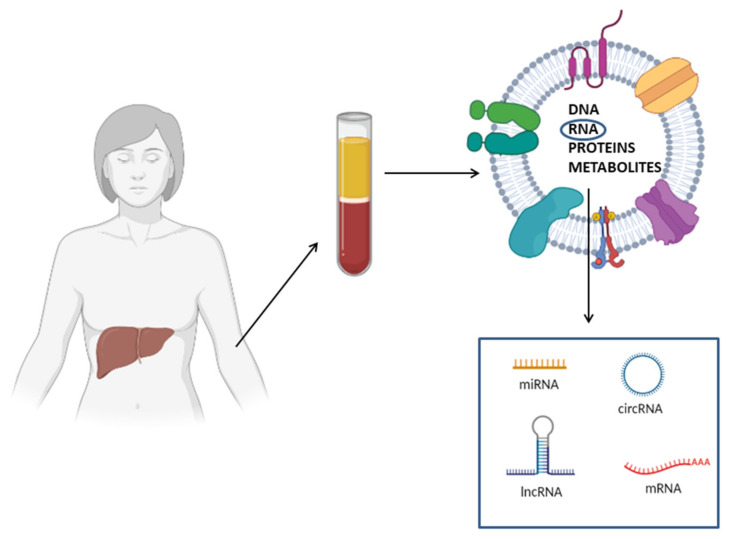
Schematic representation of circulating RNA biomarkers for NAFLD diagnosis. Circulating RNAs (e.g., mRNAs, miRNAs, lncRNAs and circRNAs) can be embedded in membranous vesicles, associated with RNA binding protein or in a free form. Membranous vesicles, besides RNA, contain other macromolecules, including DNA, proteins and metabolites.

A key feature making cf-RNAs excellent biomarkers is their high stability: these molecules are protected by endogenous RNases and are resistant to different external insults, such as multiple thaw–draw cycles, boiling, extreme PH conditions, prolonged storage and exogenous RNase treatment [79,80]. Circulating RNAs are extremely stable because they are not present in a free form in the circulation; instead, they are encapsulated in membranous vesicles (micro-vesicles, exosomes, apoptotic bodies), or alternatively complexed to RNA binding proteins (i.e., NPM1, Argonauta 2) or associated with lipoproteins. All these mechanisms protect RNAs from degradation [81]. Another aspect making RNA molecules optimal biomarkers is that the techniques used for their detection are extremely sensitive. Differently from proteins, nucleic acids are detected and quantified through PCR-based methods that determine an exponential increase of the initial template. Consequently, protein detection techniques are much less sensitive with respect to methods for nucleic acid detection. In theory, even a single RNA molecule could be detected through quantitative PCR [82]. Finally, it is important to highlight that several studies reported that the differential expression profiles of circulating RNAs are correlated to different physiological and pathological states [83]. These studies support the use of RNA molecules as non-invasive biomarkers; furthermore, their whole expression profile can be analyzed through high-throughput methods, including next-generation sequencing (NGS) and microarray, and thus, analysis of these molecules has a key role in biomarker discovery [82] (Figure 3).

### 5.1. Circulating RNAs as NAFLD Biomarkers in the General Population

Most of the studies concerning circulating RNAs as NAFL biomarkers are limited to miRNAs. Akuta et al. analyzed miR-122 expression levels in NAFL patients who underwent two serial biopsies. They observed that, in patients showing an improvement of their histopathological scores, serum miR-122 expression levels decreased at the second biopsy with respect to the first biopsy. Furthermore, there was a significant correlation between miR-122 expression levels and histopathological score variations. The authors also observed an association between miR-122 expression levels and routine clinical parameters such as AST and ALT [84]. A study reported that miR-21 levels were also decreased in NAFL patients with respect to controls [85]. Yamada et al. analyzed the expression of 5 miRNAs involved in lipid homeostasis (miR-21, -34a, -122, -145, -451) in a total of 403 subjects. Serum levels of four of these miRNAs were higher in NAFL subjects with respect to controls (92/403) (miR-21, -34a, -122, -451); furthermore, miR-122 expression levels correlated with steatosis grades [86].

In another study, more advanced techniques were used; through deep sequencing, miRNA expression profiles were evaluated in 20 NAFL patients and 20 controls. Subsequently, differentially expressed miRNA were validated in both a wider cohort (90 controls versus 152 NAFL) and an external cohort (80 controls versus 103 NAFL). The analyzed miRNA panel (miR-122, -1290, -27b, -192) showed a high NAFL diagnostic accuracy (AUC: 0.86, sensitivity = 85.55%, specificity = 73.3%). Furthermore, their sensitivity and specificity was superior to ALT and FIB-4 [87]. Raitoharju et al. demonstrated that blood miR-122 and -885 were upregulated in a cohort of 871 subjects with ultra-sonographically diagnosed fatty liver. MiR-122 and miR-885 were upregulated in NAFLD subjects and miR-122 diagnostic performance was similar to transaminase diagnostic performance. Moreover, through the metabolomic approach, they demonstrated that miR-122 expression was positively correlated with some lipoprotein classes (VLDL, IDL, LDL), while miR-885 expression was inversely associated with HDL cholesterol [88]. Another study reported that miR-181d, -99a, -197 and -146b expression levels were lower in NAFL subjects with respect to controls; furthermore, miR-197 and -146b levels negatively correlated with inflammation grade, while miR-181d and -99a levels were inversely associated with GGT in NASH patients [89]. Finally, recent studies reported that miR-29, -1296, -132 and -135 were deregulated in NAFL patients and correlated with the risk of disease onset [90,91,92,93,94]. Only one study, instead, evaluated lncRNAs as an NAFL biomarker. Long-noncoding RNAs are transcripts longer than 200 nucleotides lacking a long protein-coding open reading frame (ORFs) that are involved in a myriad of cellular processes through the regulation of gene expression at epigenetic, transcriptional, post-transcriptional, translational and post-translational levels [95]. The ARSR lncRNA has been reported to be upregulated in serum of NAFL patients versus NASH patients; furthermore, in NASH in vivo models, it has been demonstrated that this lncRNA is involved in hepatic lipogenesis [96].

**Figure 4:**
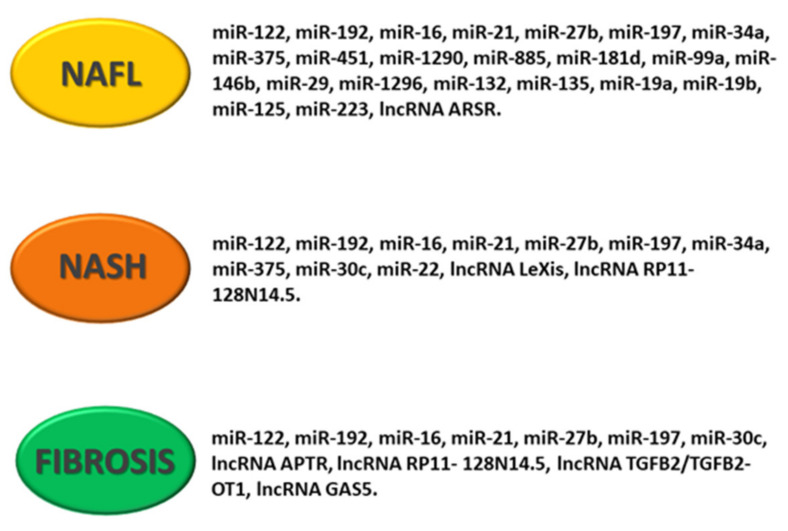
Schematic representation of deregulated noncoding RNA pattern in NAFLD. The figure shows specific miRNAs and lncRNAs associated with NAFL (non-alcoholic fatty liver), NASH (non-alcoholic steatohepatitis) and hepatic fibrosis.

Circulating extracellular vesicles (exosomes and ectosomes) containing miRNAs, mRNAs, proteins and DNA molecules could be used as NAFLD biomarkers [12]. Ectosomes are vesicles of various size (0.1–1 mm in diameter) that bud directly from the plasma membrane and are shed to the extracellular space [97]. Once released, ectosomes bind to recipient cells and deliver their informative cargo. In NAFL subjects, an increase in ectosomes in monocyte and natural-killer cell surfaces and a decrease in neutrophyl and endothelian cell surfaces have been observed. Production of exosomes and other extracellular vesicles are increased in patients with NASH [98], and it has recently been suggested that a specific protein signature in serum extracellular vesicles may be used to diagnose NASH non-invasively [99] (Figure 4).

### 5.2. Circulating RNAs for Differential Diagnosis between NAFL and NASH

As highlighted for NAFL, and also as far as NASH is concerned, most data concerning circulating RNAs are related to miRNAs. The first attempt to identify circulating miRNAs, such as NAFL/NASH differential diagnosis biomarkers, was conducted by Cermelli et al. in 2011. In more detail, they reported an increase of miRNA-122, -16 and -34a expression levels in 34 NAFLD patients with respect to 19 controls. On average, miR-122 presented a 7.2-fold change upregulation, miR-34a presented a 5.3-fold change upregulation and miR-16 was undetectable in control samples, while pathological samples had 1000 copies per mL [100]. The results obtained sub-stratifying the NAFLD population are more interesting; through this analysis, the authors found that miRNA-122 and -34a further increased in NASH patients with respect to simple steatosis patients, with a fold change of about two and three, respectively. Moreover, through ROC curve analysis, it has been demonstrated that miRNA-122 and -34a have a high diagnostic performance (miRNA-122 AUC: 0.93; miRNA-34a AUC: 0.96) in distinguishing NAFLD patients versus controls, but this decreased in the NASH versus NAFL comparison (respectively, 0.70 and 0.76) [100]. 

Another important study conducted by Pirola et al. in 2015 aimed to identify NAFL versus NASH differential diagnosis biomarkers. They profiled the expression of 84 miRNAs in patients with NAFL, NASH and controls (n = 48), and successively, they confirmed the upregulation (FC > 2) of a six-miRNA panel in an independent cohort (n = 96): miR-122, -192, -19a, -19b, -125 and -375. Only three of them, miR-122, -192 and -375, correlated with histological severity and were significantly overexpressed in NASH patients with respect to NAFL patients (AUC: 0.69, 0.68 and 0.72 respectively, in NAS > 5 versus NAS < 5 discrimination) [101].

miR-122 and -192 were further validated as indexes of histological severity by Becker et al. [102].

In more detail, they analyzed the expression of four miRNAs, i.e., miR-122, -192, -21 and -223, in 137 NAFLD patients with moderate or severe obesity (87 NASH and 50 simple steatosis (SS)) compared to 61 controls. Furthermore, they performed correlation analysis between miRNA expression level and routine biomarkers such as AST, ALT and CK-18-Asp396 fragments (released by hepatocytes during apoptosis).

Among the analyzed miRNAs, miR-122 and miR-192 were upregulated in NASH patients with severe obesity with respect to SS patients with severe obesity. However, this deregulation was not confirmed in the NASH/SS comparison in patients with moderate obesity. miR-21 was upregulated in NASH patients with respect to SS patients with moderate or severe obesity. miR-223 was upregulated in NASH patients with respect to controls, but it was not able to discriminate between the two pathological conditions [102].

Moreover, the expression levels of miR-122 and -192 had a positive correlation with ALT, CK-18-Asp396 and the NAS score, and miR-21 positively correlated with ALT and the NAS score. Subsequently, serum concentrations of these microRNAs have been used to elaborate a scoring system ranging from 0 to 3 in order to determine the diagnostic performance for NAFL versus NASH discrimination. Through these analyses, Becker et al. found that the combination between the miRNA-based scoring system and CK-18-Asp396 reached an AUC of 0.83, with a sensitivity of 93% and a specificity of 83% [102].

In a subsequent study [103], Liu et al. observed an increase of miR-122, -192, -34a and -16 expression in 48 biopsy-proven NAFLD patients with respect to 37 controls; among these miRNAs, miR-122, -16 and -34a were able to discriminate NASH (n = 31) versus NAFL (n = 17) patients. It is important to highlight that miR-34a had a higher diagnostic power and sensitivity for NASH (AUC: 0.81, sensitivity: 0.70, specificity: 0.86) than other already known biomarkers, such as ALT (AUC: 0.68, sensitivity: 0.84, specificity: 0.94), CK-18-M30 and CK-18-M60 (M30 AUC: 0.70, sensitivity: 0.41, specificity: 0.94; M60 AUC: 0.72, sensitivity: 0.52, specificity: 0.94).

Finally, a more recent study concerning miRNAs as progression biomarkers of hepatic steatosis was conducted in 2018 by Lopez-Riera et al. Analyzing miRNAs already reported to be deregulated in NAFLD, they demonstrated that miRNA-27b, -34a, -22, -122 and -192 and miRNA-30c, -16 and -197 are reduced in the serum of patients with severe disease with respect to simple steatosis patients. Furthermore, miR-34a/mir-197 and miR-27b/miR-30c ratios showed the best diagnostic performance for the identification of NASH, defined as SAF ≥ 2 (AUC: 0.81) or NAS score ≥ 5 (AUC: 0.79) [104]. Therefore, to date, based on the above-reported data, we can affirm that the upregulation of miRNA-122, -192 and -34a is consistent in several studies. Further validation in larger cohorts could lead to the introduction of these RNA-based biomarkers in clinical practice for the identification of patients with a high risk for NAFL to NASH progression. 

Few studies evaluated lncRNAs as NASH biomarkers. Park et al. analyzed the expression level of Lexis lncRNA in plasma samples of 35 biopsy-proven NASH patients versus 9 NAFL patients. They found that Lexis was increased in NASH patients versus NAFL patients, with AUC: 0.74, sensitivity: 54.3% and specificity: 100% [105].

In a study published in 2019 by our research group [106], after microarray analysis, the upregulation of RP11-128N14.5 lncRNA was validated in patients with severe NAFLD (NAS score ≥ 5 versus NAS score ≤ 4), with a diagnostic power of 0.71 (sensitivity = 73.7%, specificity = 70.4%) superior to AST (AUC = 0.66) and ALT (AUC = 0.37) (Figure 4).

### 5.3. Circulating RNAs for Fibrosis Stage Definition

It is widely known that miRNAs are involved in NAFLD pathogenesis and their deregulation has been reported in NASH-related fibrosis [107]. Although there are many studies reporting miRNA roles in pathogenesis, very few studies evaluated circulating miRNAs as biomarkers of fibrosis in NAFLD populations. 

The expression of miR-122 has been analyzed in 67 NAFLD patients with several degrees of fibrosis. miR-122 levels were significantly lower in patients with severe fibrosis versus mild fibrosis. The diagnostic performance of miR-122 was also evaluated and compared with other fibrosis biomarkers, such as hyaluronic acid and type IV collagen. miR-122 had a stronger diagnostic accuracy (AUC: 0.82) versus hyaluronic acid and type IV collagen [108].

On the contrary, Pirola et al. demonstrated that miR-122 was upregulated in patients with advanced fibrosis F2–F3 versus patients with fibrosis stages F0–F1, with an AUC of 0.61. Although this value is not high enough for a biomarker, it is important to highlight that miR-122 efficiency was higher than CK-18 (AUC: 0.49), ALT (AUC: 0.59) and AST (AUC: 0.64) in fibrosis prediction [101]. 

In 2018, López-Riera et al. [104] analyzed the expression of a set of miRNAs previously reported to be deregulated in NAFLD. miRNA expression was analyzed stratifying the patient cohort according to fibrosis stages in a two-group comparison (F ≤ 2 or F > 2) or in a five-group comparison (F0, F1, F2, F3, F4, F5). Through this latter comparison, they observed that miR-122 and miR-192 had an increased expression trend in mild fibrosis (F1 and F2) and a decreasing expression trend in severe fibrosis (F3 and F4). Thus, in this study, miR-122 had an opposite expression trend than in Miyaaki’s study [108], and the lack of consistency of mir-122 data limits its use as a biomarker for severe fibrosis prediction among NAFLD patients [104]. In the same study, another miRNA that had an opposite trend with respect to literature data was miR-16. López-Riera et al. [104] reported that miR-16 was downregulated in F3–F4 fibrosis; however, Liu et al. [103] reported that miR-16 was upregulated and had a good diagnostic performance in fibrosis prediction (AUC: 0.71) [103]. In addition, López-Riera et al. reported that circulating miRNA-21 and -27b are upregulated, while miRNA-30c is downregulated in F ≤ 2 versus F > 2 fibrosis patients. However, diagnostic performances of the analyzed miRNAs for severe fibrosis identification did not overcome conventional fibrosis algorithms. The best-performing miRNA was miR-30c, showing an AUC value of 0.72; moreover, the miR-27b plus miR-30c combination and the miR-27b plus miR-197 combination slightly increased the diagnostic performance, reaching AUC values of 0.770 and 0.75, respectively [104].

To date, few studies have taken into account the dosage of lncRNAs as NASH-related liver fibrosis biomarkers. Yu et al. demonstrated that APTR (Alu-mediated p21 transcriptional regulator) serum levels were upregulated in 34 biopsy-proven cirrhosis patients with respect to 24 controls [109]; thus, APTR could represent a potential biomarker of NASH-related hepatic cirrhosis.

In our previously published study in 2019 [106], after microarray analysis, we validated the upregulation of serum TGFB2/TGFB2-OT1 lncRNA in F3–F4 stages with respect to F0–F2 patients. TGFB2/TGFB2-OT1 lncRNA showed an AUC value of 0.80 in F3–F4 versus F0–F2 discrimination (sensitivity = 65%, specificity = 81.3%). In addition, when TGFB2-OT1 expression levels were combined with FIB-4 or FibroScan, there was an improvement in diagnostic performance. 

Finally, Han et al. [110] analyzed the expression of GAS5 lncRNA in plasma samples of patients with several fibrosis stages. GAS5 plasma levels were increased in patients with advanced fibrosis with respect to patients with absent or moderate fibrosis (F < 2), and the expression of GAS5 positively correlated with several fibrosis stages (from F0 to F3). However, GAS5 was upregulated in F3 versus F < 2, and its expression was downregulated in NAFLD patients with cirrhosis (F4) versus patients with advanced fibrosis (F3). This is the first study that demonstrated that GAS5 levels are altered during fibrosis progression and cirrhosis development (Figure 4).

Our study provides novel high-throughput data concerning the expression of non-coding RNA in serum of NAFLD patients and lays the foundation for the identification of novel biomarkers that could represent a future alternative to hepatic biopsy.

## 6. Conclusions

To date, NAFLD is a risk for global health because of its morbidity, mortality and high prevalence in the general population [111].

The possibilities to correctly identify NASH patients among NAFLD subjects and to stage fibrosis are important clinical challenges. Currently, hepatic biopsy is still the gold standard to diagnose NASH and stage fibrosis. However, because of risks related to biopsy, clinicians often use biochemical and imaging tests, even though they are characterized by limited diagnostic performance [13,14,21]. The identification of novel non-invasive methods is fundamental because it could make the monitoring of treatment response and disease progression easier. Over the last few years, several classes of molecules such as specific proteins or metabolites have been proposed to diagnose NAFL, NASH and stage fibrosis. Their limited diagnostic performance restricted the clinical application of these markers for NAFLD screening and fibrosis staging. Nevertheless, it is important to highlight that several studies demonstrated that the combination of several serum biomarkers in panels increased their diagnostic performance. Therefore, this combined approach could allow, in the future, clinical application for steatosis diagnosis and staging.

The introduction of novel “omics” technologies could allow the identification of novel non-invasive biomarkers for NAFLD patient identification, NASH versus NAFL patient discrimination and fibrosis staging. Nevertheless, these “omics studies” need to be validated in larger and more heterogeneous cohorts before being used in clinical practice.

## Data Availability

Not applicable.
